# Design considerations for a theory-driven exergame-based rehabilitation program to improve walking of persons with stroke

**DOI:** 10.1007/s11556-013-0136-6

**Published:** 2013-12-07

**Authors:** Seline Wüest, Rolf van de Langenberg, Eling D. de Bruin

**Affiliations:** 1 Department of Health Sciences and Technology, Institute of Human Movement Sciences and Sport, Wolfgang-Pauli-Str. 27, 8093 Zurich, Switzerland; 2 ETH Zurich, Zurich, Switzerland

**Keywords:** Stroke rehabilitation, Gentile's taxonomy, Virtual reality, Exergames, Motor learning

## Abstract

Virtual rehabilitation approaches for promoting motor recovery has attracted considerable attention in recent years. It appears to be a useful tool to provide beneficial and motivational rehabilitation conditions. Following a stroke, hemiparesis is one of the most disabling impairments and, therefore, many affected people often show substantial deficits in walking abilities. Hence, one of the major goals of stroke rehabilitation is to improve patients' gait characteristics and hence to regain their highest possible level of walking ability. Because previous studies indicate a relationship between walking and balance ability, this article proposes a stroke rehabilitation program that targets balance impairments to improve walking in stroke survivors. Most currently, available stroke rehabilitation programs lack a theory-driven, feasible template consistent with widely accepted motor learning principles and theories in rehabilitation. To address this hiatus, we explore the potential of a set of virtual reality games specifically developed for stroke rehabilitation and ordered according to an established two-dimensional motor skill classification taxonomy. We argue that the ensuing “exergame”-based rehabilitation program warrants individually tailored balance progression in a learning environment that allows variable practice and hence optimizes the recovery of walking ability.

## Introduction

Virtual reality technique can be combined with targeted exergames development with the aim to promote motor rehabilitation. Recently, a number of researchers have pointed to the potential of virtual reality applications in health care [6, 20, 28, 36, 48, 50] and hence sparked the introduction of this technology within rehabilitation medicine. The use of so-called “exergames”—virtual reality games that involve physical exercise—has been proposed as a valuable instrument to encourage participation in rehabilitation and improve the adherence to therapy programs because of engaging the user [6, 12, 48]. For example, a study by Rizzo and Kim [48] demonstrated that virtual reality-based games reduce patients' dreariness and simultaneously increase their motivation for rehabilitation practice. Accordingly, virtual reality provides the capacity to simulate scenarios that are effective in attracting the performers' attention. Simulated circumstances can be used to elicit a thrilling ambience, whereas the patient can still perform movement and behaviors in a safe and controlled environment.

For best results in rehabilitation, video games specifically designed for therapy should be used [5]. Because conventional video games were primarily developed for entertainment purposes [63], most of these are not practical for rehabilitation [5]. One hurdle facing the successful use of exergames in rehabilitation is that many off-the-shelf video games are too complex for use by functionally impaired persons or elderly people [12]. Video games must therefore be developed to take into consideration the cognitive and physical limitations, as well as the interest sets, of the trainees [12]. To date, however, the vast majority of research has focused on games developed for the entertainment market which fails to adapt the gameplay according to patients' rehabilitation requirements [44].

FP7 is the short name for the Seventh Framework Programme for Research and Technological Development, the European Union's main instrument for funding research in Europe running from 2007 to 2013. FP7 is also designed to respond to Europe's employment needs, competitiveness, and quality of life [c.f. http://ec.europa.eu/research/fp7/index_en.cfm?pg=understanding]. The research project “*Rehabilitative Wayout In Responsive home Environments”* (REWIRE) [46], which is funded by FP7, aims to develop, integrate, and field test an innovative virtual reality-based rehabilitation platform for people with stroke. The platform should allow patients, discharged from the hospital, to continue intensive rehabilitation at home under remote monitoring by the hospital. The main idea is to combine off-the-shelf components (e.g., the tracking device Microsoft Kinect [30], the force plate Tymo [58]) in a robust and reliable way and render a system that can be used in the stroke patients' homes. In the context of the REWIRE project, the need has been recognized for exergames specifically targeted at walking rehabilitation [5].

When creating a virtual reality-based stroke treatment approach, the development of suitable exergames is undoubtedly an important requirement. There have been, to date, some studies that deal with the development of video games adequate for therapy purposes. Based on specific game design principles, they intend to provide engaging and challenging treatment conditions to achieve a high level of motivation for rehabilitation practicing [1, 6, 7, 24, 33, 43, 59]. Moreover, it is important that the developed exergames are functionally integrated in a well-elaborated therapy program. On the one hand, the rehabilitation program should be aimed at appropriate rehabilitation goals. That is, it should be aimed at those goals that are considered to be essential for (and by) stroke victims. On the other hand, the rehabilitation program should be consistent with established training principles to successfully reach these goals. In this article, we describe how tailored exergames for stroke patients can be developed based on a theoretical framework. We will discuss key exergame design and training program content considerations for patients with stroke, which may be theoretically linked to impaired walking performance due to the stroke event.

## Stroke-induced motor impairments and their treatment approach

Worldwide, there are about 4.8 million survivors of stroke of whom about 1.1 million suffer lasting functional disabilities [14]. The specific disabilities caused by stroke vary greatly depending on the brain area that is damaged. Hemiparesis—a paralysis that characteristically affects an arm and leg on one side of the body—is one of the most common stroke-induced impairments [34] and often leads to a number of negative walking-related consequences. The typical “hemiparetic gait” post-stroke is associated with a reduced walking velocity, cadence and stride length, with gait asymmetry, and with a prolonged double-support and stance-phase duration of both lower extremities [15, 26, 27, 45, 61]. It is for this reason that many stroke victims show marked deficits in walking abilities [51]. There is general agreement that an association exists between the degree of independent mobility and quality of life [47, 54, 56]. Consequently, a central goal of stroke rehabilitation should be to improve gait characteristics and to retrain the patient to the highest possible level of walking ability [16, 34]. The findings demonstrated by Vincent et al. [60] confirmed the importance of enhancing stroke patients' functional mobility through rehabilitation. Vincent and colleagues [60] set out to reveal rehabilitation needs from the perspective of four different parties involved in the stroke rehabilitation process (stroke patients, caregivers, health professionals, and healthcare managers) so as to better plan the post-stroke treatment service. According to patients' statements—which, in our view, should have high priority when designing rehabilitation programs—continued rehabilitation should focus primarily on motor activities, such as walking. Patients' statements thus further highlight the importance of gait recovery.

Good balance skills are an important determinant of walking performance and impaired balance ability is assumed to be related to a decreased locomotor function [18, 62]. Considering that hemiparesis not only affects gait characteristics, but also often leads to diminished balance skills, a post-stroke rehabilitation program targeting deficits in static and dynamic balance may be an effective way to restore independent functional walking. In recent years, another factor contributing to walking recovery has been suggested: Several authors noted that task-related activities lead to greater improvements in post-stroke walking competency than non-task-related practices [13, 42, 49]. Specifically, they suggested that intervention protocols that include actual walking tasks improve walking skills to a greater extent than rehabilitation programs that do not. Additionally, there is robust evidence in the motor learning literature that the best outcomes in terms of long-term retention and transfer of skills are achieved when principles of motor learning are integrated into treatment protocols. In rehabilitation, it is widely accepted that more practice is better and that an intense structured therapy program with numerous repetitions of various, challenging tasks supports motor skill acquisition [38]. Specifically, two important elements in our consideration are the motor learning principles *variable practice* and *progression*. These principles are known to positively affect gait rehabilitation and, hence, should be considered in developing a rehabilitation program.

The literature to date has found that variable practice leads to better transfer and retention of motor skills than if a constant practice structure is used [22, 23, 31, 38, 52]. Instead of performing one task repeatedly and always in the same manner (constant practice), a specific task should be practiced differently throughout a treatment session by varying the conditions of practice (variable practice) [38]. Krakauer [31]—in his contribution published in the *Current Opinion in Neurology*—stated that variable practice is a fundamental principle in terms of retaining learning over time and that a consistent agreement in the literature exists indicating that varied practice is superior to repetitive identical tasks when it comes to motor learning. The principle of progression holds that motor learning and rehabilitation programs benefit from a continuous adaptation of task difficulty to increasing skill level [19]. For successful learning, an optimal challenging training situation should be given providing an appropriate task difficulty level according to individuals' capacities [17]. Furthermore, it can be hypothesized that the training effects will be enhanced when the locomotor practice gets combined with direct feedback; e.g., visual feedback. Visual feedback can be used to provide information about a patient's movement or the result of a movement and is known to promote postural control and stability [2, 19, 25, 53, 62].

From the above, it follows that rehabilitation programs for stroke survivors are well-advised to train balance and walking skills by means of a variety of balance- and walking-specific exercises, the difficulty of which is progressively adapted to patients' skill level, in combination with direct visual feedback on performance.

Although previous studies have emphasized the importance of theory-based practice and rehabilitation [3, 11, 37, 57], there is still a lack of established concepts underlying the practical implementation of motor skill learning principles and theories in rehabilitation. A desirable template would be one that is simple and feasible and that facilitates the task-specific, progressive, and variable training of balance and walking skills. The motor skill taxonomy proposed by Gentile [21] seems to constitute just such a template for rehabilitation programs because it provides a two-dimensional basis for classifying a variety of motor skills. Based on the above, the aim of this article is to develop and describe a tailored exergames-based stroke rehabilitation program that is based on a theoretical framework.

## Methods

Four prevalent classification systems exist for identifying common characteristics of motor skills. Three of these categorize motor skills according to one common characteristic of the skill and lead to one-dimension classification systems. In contrast, Gentile's taxonomy considers two general skill characteristics and offers, therefore, a broader concept leading to a two-dimensional classification system of motor skills. To highlight the high potential of Gentile's two-dimensional approach, it is worthwhile to focus at first on the one-dimensional classification systems and discus some of their limitations:

A description of these classification systems is given by Magill [35] (pp. 5–16). One one-dimension classification system differentiates skills depending on the sizes of the primary muscle groups required to produce an action (gross motor skills vs. fine motor skills). A second one-dimensional system considers the specificity of a movement's beginning and end points to categorize motor skills (continuous motor skills vs. discrete motor skills). The third one-dimension classification system makes a distinction according to the stability of the environmental context in which an action is being performed (open motor skills vs. closed motor skills) [35, 39]. These one-dimensional classification systems raise the problem that they fail to capture the complexity of many motor skills [35] by only focusing on a single aspect of motor skills.

## Gentile's motor skill taxonomy

Gentile presented a systematic classification system to categorize motor skills and movement according to two general dimensions of physical actions [32]. The first dimension, *environmental context*, refers to the environmental conditions to which the performer has to react in order to successfully perform a task. This dimension is characterized by two indicators: (a) *regulatory conditions* and (b) *intertrial variability*. The *regulatory conditions* indicate relevant environmental features that constrain movement execution and may either be stationary (*stationary regulatory conditions*) or moving (*in-motion regulatory conditions*). With the indicator *intertrial variability*, Gentile's taxonomy differentiates between regulatory conditions that change between trials (*intertrial variability*) and those that do not (*no intertrial variability*). The second dimension, *action function*, is also characterized by two indicators: (a) *body orientation* and (b) *object manipulation. Body orientation* indicates whether an action requires the performer to move from one location to another (*body transport*) or not (*body stability*). *Object manipulation* indicates whether an object has to be controlled during the action performance (*object manipulation*) or not (*no object manipulation*).

Through the interaction of the resulting four environmental context characteristics and four action function characteristics, Gentile defines 16 different motor skill categories that provide a comprehensive template to classify motor skills (Table 1). The taxonomy is a good means of becoming aware of the skill characteristics that make skills distinct from, as well as related to, other skills, and is an excellent guide for establishing practice or training routines [35].

**Table 1:**
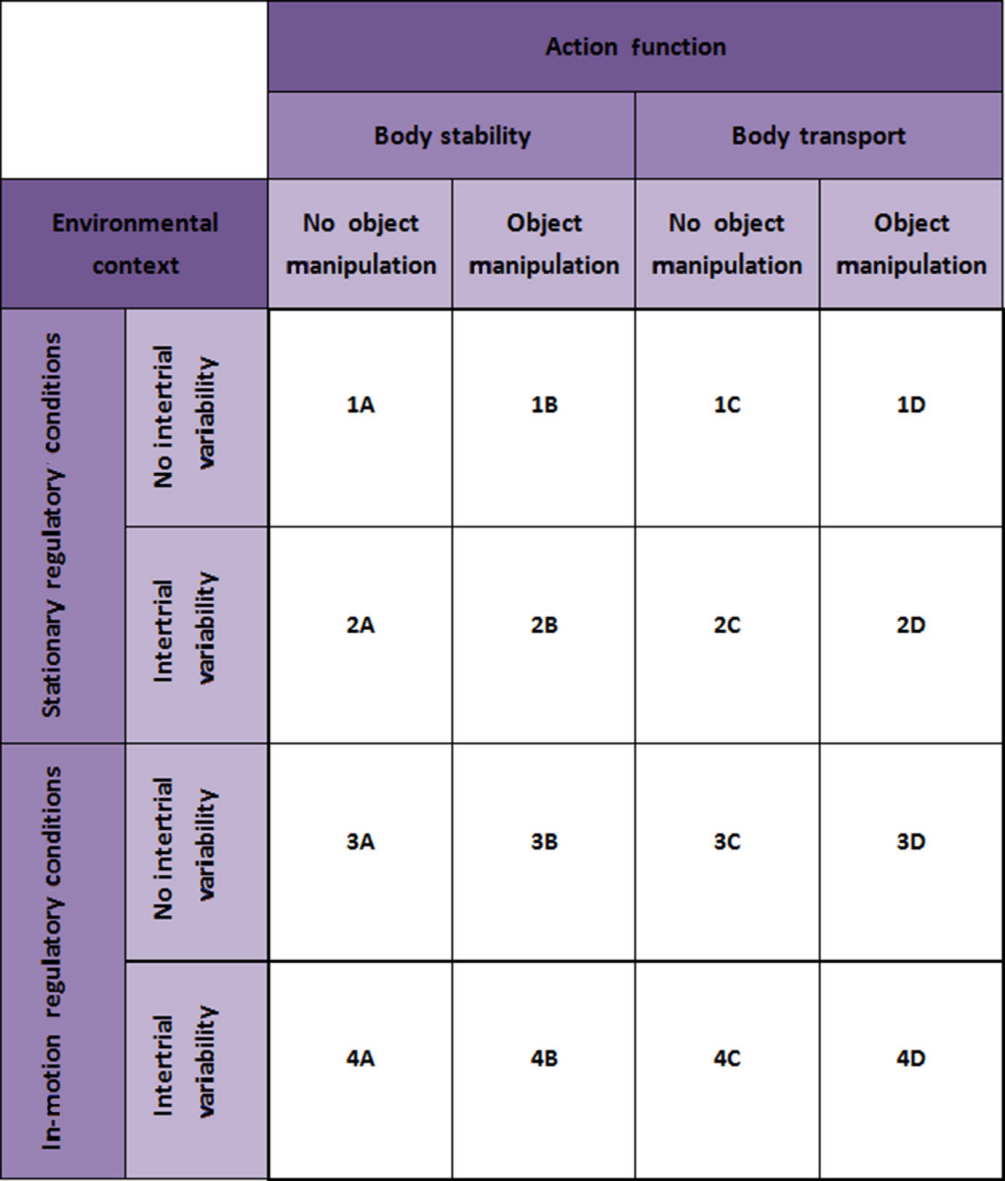
Gentile's taxonomy of motor skills [35]

According to Gentile, the easiest skill category can be found at the top left position (1A). Moving either rightward or downward in the table renders the skill category more difficult, so that the most difficult skill category can be found at the bottom right of the table (4D). For the action function dimension, this implies that body transport is more difficult than body stability and object manipulation more difficult than no object manipulation. Importantly, it also assumes a hierarchy in the action function characteristics: skills involving body transport but no object manipulation are more difficult than those involving object manipulations but not body transport. For the environmental context dimension, the same pattern of progression is assumed.

Thus, Gentile's taxonomy allows a systematic progression in difficulty of motor tasks and meets the demand of the motor learning principle *progression*. Moreover, each of the 16 categories is associated with unique features based on the two-dimensional approach and, consequently, the taxonomy involves task variations. Obviously, Gentile's classification system is consistent with the motor learning principle *variable practice*.

There are several reasons for assuming that this taxonomy provides a valuable tool for developing a theory-based rehabilitation program using virtual reality. Considering the explanations above, it is evident that Gentile's taxonomy is in accordance with the generally accepted motor learning principles *progression* and *variable practice*. The taxonomy provides a well-structured framework and the 4 × 4 table can serve as a useful guide for preparing a systematically coherent motor learning concept in a simple manner. For implementing Gentile's taxonomy-based approach, exergames might demonstrate an optimal opportunity for providing beneficial treatment conditions. On the one hand, as we referred above, there is high potential for creating a motivating rehabilitation environment using virtual reality. On the other hand, the taxonomy seems suited for virtual reality applications where the environment, task complexity, and other contextual factors related to exercise performance can be manipulated. Exergame parameters can be easily modified and, thereby, adjusted to the demands of the taxonomy-included skill categories.

## Gentile's taxonomy-based exergames to improve stroke patients' balance and walking skills

To provide a post-stroke rehabilitation program focused on improved balance skills in standing and walking in accordance with Gentile's framework, we designed six basic exergames. Based on these six basic exergames, we created 16 virtual reality scenarios, either by making game parameter modifications or by substituting another basic game. Each scenario corresponds to one of the 16 skill categories included in the taxonomy (Table 2).

**Table 2:**
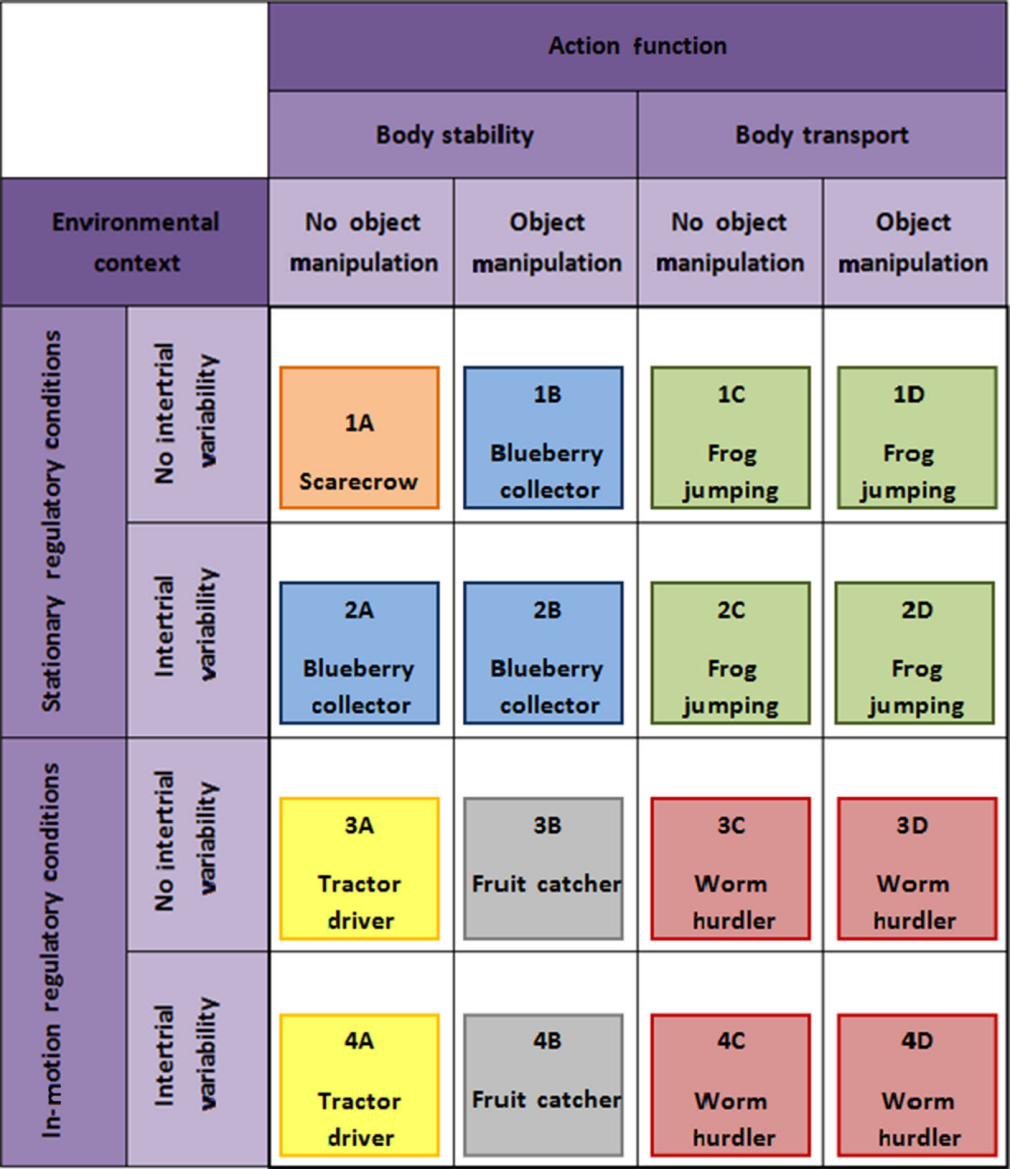
Exergame scenarios corresponding to Gentile's skill categories

In accordance with a phased iterative approach suggested by Campbell et al. [8], this paper demonstrates the theoretical phase—the first step—when developing and evaluating complex research-based interventions to improve health. Thus, a set of six basic exergames appears to be appropriate in the research process of designing an exergame-based stroke rehabilitation program. It keeps the number of games that have to be developed manageable and—with the same game being implemented across adjacent skill categories—it renders systematic progression more straightforward.

Below, we will briefly describe the content of each of the 16 skill categories.

1. The first phase of the rehabilitation program focuses on the basic physical skill of standing quietly. In this exergame, the patient is represented as a scarecrow avatar and is required to maintain a cursor indicating the body's center of pressure (COP) within a predefined marked circle on a computer screen [40]. The exergame focuses on stance steadiness (sway) that demonstrates a relevant aspect related to an individual's balance ability [40]. Due to hemiparesis after stroke, patients often show an increased amount of postural sway [40].
2. Whereas the game *Scarecrow* described for skill category 1A demands a centered and stable COP position, this game (called *Blueberry Collector*) uses controlled COP displacements for successful performance. This exergame targets stroke patients' dynamic stability, because subjects with hemiparesis often suffer from reduced limits of stability [10]. The virtual environment represents a field of blueberries, which have to be collected by a virtual farmer. The farmer's moving is controlled by the patient's weight shifting tasks and, in this way, the blueberries can be gathered. Because the position of the berries in the field is fixed and unvarying between the different exergame trials, *stationary regulatory conditions* are given and *no intertrial variability*. To meet the requirement of *object manipulation*, a virtual basked filled with blueberries is placed on the farmer's head. In order to prevent berries from falling down, the patient must adopt an ideal upright body posture.
3. Previous exercise interventions have shown that stepping exercises indicate an effective treatment method to improve postural stability and balance [9, 29, 41], Accordingly, this exergame (called *Frog jumping*) requires the control of the COP displacements during stepping movements. The virtual environment represents a pond with several stones placed in it. By doing steps in a specific direction, the patient can control a frog jumping from one stone to another. A virtual arrow indicates which particular stone has to be targeted by the patient. In accordance with the taxonomy conditions, the position of each stone within the pond is fixed (*stationary regulatory conditions*) and the order of the targeted stones unchangeable from one exergame trial to the next (*no intertrial variability*).
4. According to Gentile's taxonomy, the skill category 1D can be seen as a progression of 1C. Thus, the game *Frog jumping* described for 1C can be extended by integrating an o*bject manipulation* task. Specifically, in this game, the virtual frog is wearing a crown and it is the patient's task to perform stepping tasks with an upright body position in order to keep the virtual crown correctly positioned.
5. The game *Blueberry collector* described above is not only adequate for skill category 1B, but also for category 2A by making small adaptations. On the one hand, the virtual basket on the farmer's head has to be removed to get the condition *no object manipulation*. On the other hand, because *intertrial variability* is required, the position of the berries varies within exergame trials.
6. For skill category 2B, the exergame *Blueberry collector* for category 2A can be extended with an *object manipulation* task. Thus, the virtual farmer is balancing a basket on his head such as in the exergame described for skill category 1B.
7. We use the game *Frog jumping* described for category 1C with the only modification of providing a random order of targeted stones—marked by the arrow—to realize *intertrial variability*. Consequently, each time when the game has been played, a unique step performance pattern is required by the patient.
8. To meet the taxonomy requirements in skill category 2D, the game *Frog jumping* of category 2C can easily be adapted. The only modification that has to be done concerns the need for *object manipulation*. Hence, in this game, the frog is wearing a crown for which the patient has to control his/her position while playing the game.
9. For skill category 3A, we created a game called *Tractor driver*. The exergame presents a moving tractor for which the driving speed is defaulted by the video game. The direction of driving is controlled by the patient's weight shifting movements. When playing this exergame, the ability to move the COP in a standing posture without loss of balance (dynamic stability) is being trained [40]. Specifically, the purpose of this game is to fork up hay bales through directing the moving tractor from one hay bale to another. The hay bales are widely spread over the soil. Considering that the speed of the moving tractor is default and cannot be manipulated by the patient themselves, *in-motion regulatory conditions* are given. There is a fixed position of each hay bale unchangeable from one exergame trial to the next to ensure *no intertrial variability*.
10. In this exergame (called *Fruit catcher*), the patient is represented as an avatar balancing a fruit basket on the head. Virtual apples are falling down from a tree (*in-motion regulatory conditions*) that should be caught by the basket. The patient has to perform target-oriented COP shifts to get an optimal avatar position and the apples are falling into the basket. This exergame focuses on dynamic stability due to controlled weight shifting movements to selected targets. For presenting *no intertrial variability*, the game parameters are always the same, even when the game is repeatedly played.
11. According to Gentile's taxonomy, this exergame (called *Worm hurdler*) requires stepping tasks. Specifically, the patient represented as an avatar has to overstep a crawling worm coming closer alternately from the right and the left side (*in-motion regulatory conditions*). The parameters of the game are unchangeable and, thus, *no intertrial variability* is given.
12. The exergame *Worm hurdler* described in 3C can easily be modified for skill category 3D. By integrating a virtual water jar that has to be balanced on the avatar's head, an *object manipulation* task is integrated. To avoid water spillage, the patient has to adopt an ideal upright body posture.
13. For skill category 4A, we adapted the game *Tractor driver* described for category 3A by implementing *intertrial variability*. Specifically, to provide varying regulatory conditions, the hay bale positions change from one exergame trial to another.
14. The exergame *Fruit catcher* described for skill category 3B needed a slight modification to meet the category requirements for 4B. In this game, there are not only falling apples which have to be caught, but various kinds of fruits. On the one hand, the fruits have different sizes and, on the other hand, different falling speeds. Obviously, *intertrial variability* is given.
15. When playing the game *Worm hurdler* designed for category 3C, but with changing regulatory conditions between the exergame trials, *intertrial variability* is ensured. Accordingly, in this game, the worms are crawling with varying speeds and are coming either from the right or the left side in random order.
16. For integrating an *object manipulation* task, the exergame *Worm hurdler* described for skill category 4C can be advanced by balancing a virtual water jar on the avatar's head. Consequently, while overstepping the randomly approaching crawling worms, the patient has to adopt an ideal upright body position ensuring that no water is spilling out (from the jar).

## Practical application of the taxonomy-based rehabilitation program using exergames

When implementing a therapeutic program to improve stroke victims motor skills, therapists must select exercises that are tailored to the demands of the individual patient. To achieve optimal outcomes in rehabilitation, the activities should be matched to a patient's functional abilities and limitations. In a well-elaborated training plan, the (exercise) tasks which have to be performed should maintain an optimal challenge for the patient [7, 19]. Obviously, for providing a perfectly tuned exercise difficulty level at any point in time throughout the rehabilitation period, modifying the therapy plan is an essential need and an ongoing process. When a patient is making progress, more challenging tasks should be involved into the rehabilitation program. For selecting functionally appropriate activities during rehabilitation, we highlight the practical value of using Gentile's taxonomy. Based on the consideration that the taxonomy-based skill categories present a structure going from simple to complex motor tasks, we provide a formal procedure for guiding patients from the top left of the table to the bottom right. Closer inspection of Table 1 reveals that the taxonomy can be divided into seven difficulty levels. More specifically, starting from 1 of the 16 skill categories, a category-based progression in task difficulty can be achieved either through a horizontal shift to the right or a vertical shift downward. Accordingly, seven levels of difficulty can be distinguished which define a progressive increase of complexity in diagonal direction (Table 3).

**Table 3:**
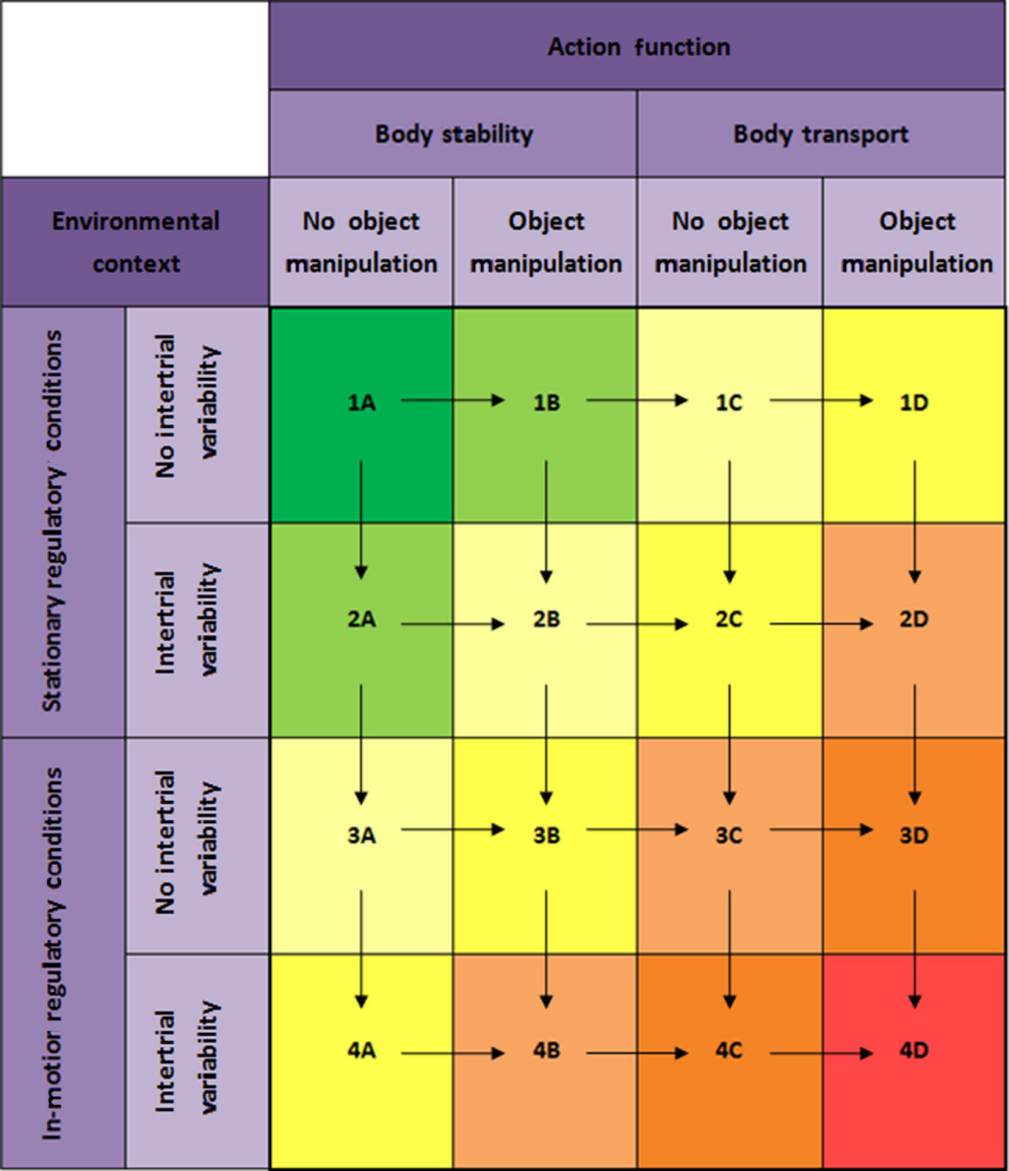
Gentile's taxonomy of motor skills divided into seven levels of difficulty (ordered diagonally)

Considering the allocation of the skill categories to one of the seven difficulty levels, Gentile's taxonomy offers a simple and easy-to-follow way for incorporating an ongoing progression into a rehabilitation process. Specifically, for selecting appropriate activities that provide rehabilitation at the optimal level of challenge, we propose the following procedure: A patient may only move up one level of difficulty when all skill categories within the current level are completed. For clarification, beginning with skill category 1A in difficulty level 1, the patient is required to move to the skill categories in difficulty level 2 (i.e., 1B and 2A) as soon as category 1A is successfully performed. Within level 2, patients and treating therapists are free to choose the order of execution of the belonging skill categories. Once a patient is capable to perform both skill categories 1B and 2A successfully, the next difficulty level 3 has to be incorporated into rehabilitation. This procedure can be continued until the patient finally achieves skill category 4D and, consequently, the highest level of difficulty (i.e., level 7). In this way, challenging situations are ensured at any point in time during rehabilitation and the patients are encouraged to exercise towards their functional limits. Following the procedure suggested to guide someone's therapy plan, one ensures that the selection of skill categories is constrained. Due to this constraint, a variation of both general dimensions defined by Gentile's taxonomy will be considered during the rehabilitation process. Accordingly, the procedure prevents a skill category-based progression only in one direction and includes the modification of both the environmental context and the action function characteristics.

Apart from these constraints, this approach also enables a certain degree of self-determination by therapy-involved actors. Within a specific level of difficulty, the performance order of the skill categories can be freely determined. This autonomy in decision-making increases the potential of tailoring the rehabilitation process to the demands of each individual patient and leaves freedom of choice to the treating therapists for the most adequate exercises that should currently be trained.

## Limitations and future directions

Using Gentile's taxonomy for designing a rehabilitation program that targets balance and walking deficits of stroke patients, an aspect must be considered critically. According to Gentile's framework, a horizontal shift to the right or a vertical shift downward within the taxonomy provides an increase of task complexity. However, when we focus on a motor task such as standing still on one leg, a potential inconsistency in Gentile's approach emerges. Standing still on one leg can be categorized by the taxonomy dimension *body stability* that represents less complex tasks than motor skills that require body transport (e.g., walking). In our view, maintaining a one-leg stance position needs good balance skills. Therefore, this activity might be perceived by some stroke patients as an advanced motor task which challenges them more than a body transport activity. Furthermore, where this theoretical approach focused on the “general stroke patient population”, we are aware of the fact that different accompanying diseases and impairments; e.g., dizziness or muscle weakness due to stroke, may cause differences in how patients perceive tasks as being more or less difficult. It can be hypothesized that some patients have more difficulty maintaining a given posture without loss of balance whereas others experience performing a body transport task as more difficult. We think, however, that these potential differences may be resolved by clinicians that treat and monitor patients and decide, together with the patients and according their skill levels, how and when to progress through the taxonomy. It is clear to us that several pilot studies assessing feasibility and usability in several reference and subpopulations are needed as necessary next step in our program development process. Feasibility studies are comparative randomized trials designed to provide preliminary evidence on the clinical efficacy of a drug or intervention [55]. Usability evaluation is a way to ensure that interactive systems are adapted to the users and should be part of a fundamental step in the user centered design process [4].

In this paper, we elucidate the theoretical basis for a theory-driven rehabilitation program to improve stroke sufferers' balance skills and walking capability using exergames. According to the framework proposed by Campbell et al. [8], investigating relevant theory prior to an exploratory trial is of great importance. Considering the theoretical knowledge gathered, an optimal study intervention design for pilot studies can be worked out that focus on the relevance and effectiveness of the rehabilitation program suggested. Depending on the usability and feasibility testing results obtained, refinements and extensions of the exergames can be made and the present set of six basic exergames may be extended to a maximum of 16 different exergames. Hence, a comprehensive and practical theory-driven stroke rehabilitation program based on Gentile's taxonomy can be achieved.

## Conclusion

With stroke, the changes in the central nervous system and lifestyle may impair an individual's ability to regain and/or maintain certain levels of motor functioning. By using virtual reality, we might be able to offer continued rehabilitation in an attractive and meaningful way. Specific targeted exergames have been designed that aim to minimize stroke-induced walking impairments. Considering the aim of presenting a well-elaborated training concept with a strong theoretical rationale, in this paper we demonstrate that Gentile's taxonomy presents a feasible template providing assistance for developing and implementing a theory-driven rehabilitation program. The motor skill taxonomy suggested by Gentile defines two general dimensions and structures a table that consists of 16 different skill categories (Table 1). On the one hand, based on these skill categories, the taxonomy provides variable training scenarios. Thus, a therapy program which meets the demand of the motor learning principle variable practice can be created. On the other hand, the taxonomy allows a systematic progression during the rehabilitation process. Accordingly, the motor learning principle of progression is explicitly considered. Due to the advantages of Gentile's two-dimensional approach, the taxonomy suits our purposes and was used as underlying framework to build up a post-stroke exergame rehabilitation program for enhancing balance and walking skills. Based on the literature reviewed here, clinical approaches to maintaining and improving physical functioning over longer time periods in persons with stroke may be combined with novel exergame-based approaches that sustain physical functioning.
